# Physical Proximity of Sister Chromatids Promotes Top2-Dependent Intertwining

**DOI:** 10.1016/j.molcel.2016.09.007

**Published:** 2016-10-06

**Authors:** Nicholas Sen, Joanne Leonard, Raul Torres, Jonay Garcia-Luis, Gloria Palou-Marin, Luis Aragón

**Affiliations:** 1 MRC Clinical Sciences Centre (CSC), Du Cane Road, London W12 0NN, UK; 2 Institute of Clinical Sciences (ICS), Faculty of Medicine, Imperial College London, Du Cane Road, London W12 0NN, UK

**Keywords:** SCI, catenation, cohesin, condensin, Top2 (Topoisomerase 2), chromosome segregation, DNA replication

## Abstract

Sister chromatid intertwines (SCIs), or catenanes, are topological links between replicated chromatids that interfere with chromosome segregation. The formation of SCIs is thought to be a consequence of fork swiveling during DNA replication, and their removal is thought to occur because of the intrinsic feature of type II topoisomerases (Top2) to simplify DNA topology. Here, we report that SCIs are also formed independently of DNA replication during G_2_/M by Top2-dependent concatenation of cohesed chromatids due to their physical proximity. We demonstrate that, in contrast to G_2_/M, Top2 removes SCIs from cohesed chromatids at the anaphase onset. Importantly, SCI removal in anaphase requires condensin and coincides with the hyperactivation of condensin DNA supercoiling activity. This is consistent with the longstanding proposal that condensin provides a bias in Top2 function toward decatenation. A comprehensive model for the formation and resolution of toxic SCI entanglements on eukaryotic genomes is proposed.

## Highlights

• Top2 introduces intertwining on chromatids independently of DNA replication
• Physical proximity between chromatids drives Top2 intertwining
• Sister chromatid intertwines are removed by Top2 at the anaphase onset
• Condensin DNA supercoiling activity correlates with Top2 intertwine removal

## Graphical Abstract

**Figure:**
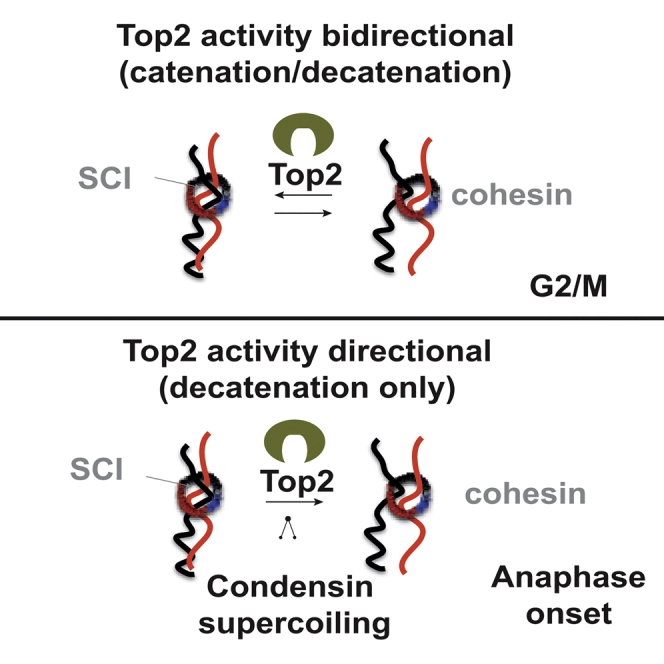

## Teaser

Sister chromatid intertwines (SCIs) are produced during DNA replication and removed by Top2 before segregation. Sen et al. show that physical proximity between sister chromatids drives Top2 to introduce SCIs in already replicated chromatids. However, in anaphase, condensin supercoiling activity provides a bias in Top2 function to remove all SCIs.

## Introduction

Unwinding of parental DNA strands by replicative helicases generates superhelical tension (in the form of positive supercoils) ahead of the replication fork. This tension can be removed either by topoisomerase-mediated relaxation of supercoils in the unreplicated parental region or by fork rotation, often referred to as fork “swiveling”, relative to the unreplicated DNA (Champoux et al., 1984). A direct consequence of fork swiveling is the formation of crosses between replicated sister chromatids behind the forks; these become sister chromatid intertwines (SCIs), or catenanes, upon replication completion. During late stages of replication, as forks converge, swiveling is thought to be the preferred pathway to eliminate superhelical tension (Postow et al., 2001). Therefore, the formation of sister chromatid intertwines (SCIs) in replicated chromosomes is thought to be a direct consequence of DNA replication (Postow et al., 2001).

Heavily intertwined sister chromatids exhibit difficulties during chromosome segregation. Cells use type II topoisomerases (like yeast Top2) to remove the bulk of SCIs between replicated chromosomes before anaphase. These enzymes work by cleaving both strands of one DNA duplex and passing a second DNA through the break before religation (Wang, 1996). When this strand-passage reaction involves cleavage of one chromatid and passage of a sister chromatid segment, the end product can be chromatid disentanglement (i.e., decatenation) or chromatid intertwining (i.e., catenation), depending on the direction of the passage. In theory, Top2 could be “blind” to the direction, and thus could introduce linkages between the sister chromatids as well as remove them. This might be a problem in the congested nuclear environment, where replicated chromosomes are woven together and sister chromatids are adjoined by cohesin complexes. Current thinking suggests that this is not an issue because Top2 action has an intrinsic bias toward decatenation. This view originates from the fact that, in vitro, type II topoisomerases decatenate plasmids below the expected thermodynamic equilibrium (Rybenkov et al., 1997). Moreover, modeling studies of Top2 action have suggested an ability to detect the chiral orientation of particular DNA crosses, leading to the proposition that Top2 is able to identify suitable substrates in a global context (Stone et al., 2003, Timsit and Várnai, 2010). Based on this, Top2 has been speculated to act as “Maxwell’s demon” (Maxwell, 1871), acting only on a subset of possible substrates (DNA crossovers resulting in decatenation) which are required to obtain a desired global topological status, i.e., full decatenation of the genome (Pulleyblank, 1997). Ergo, the accepted (although not experimentally demonstrated) view is that, inside cells, Top2 has a bias in its strand-passage activity toward decatenation. Therefore, based on our present understanding, SCIs formed during DNA replication should be removed by Top2 during the period spanning S phase and mitosis (Stuchinskaya et al., 2009). SCI removal, however, might be more complex than this prediction, because recent data suggest that SCIs persist until mitosis in yeast minichromosomes when cells are arrested with the microtubule poison nocodazole (Farcas et al., 2011, Koshland and Hartwell, 1987). Maintenance of SCIs until metaphase requires cohesion (Farcas et al., 2011); this has led to the proposal that cohesin complexes “protect” SCIs by masking them from resolution by Top2 (Farcas et al., 2011).

Condensin is not only required for metaphase chromosome condensation (Hirano, 2005), but is also necessary to prevent sister chromatids from being entangled during segregation; these two functions are likely to be linked (Koshland and Strunnikov, 1996). Therefore, it has been proposed that chromosome condensation, and condensin itself, might somehow facilitate Top2’s role in removing SCIs from anaphase chromosomes (Koshland and Strunnikov, 1996, Nasmyth, 2001). Pioneering in vitro studies demonstrated that condensin overwinds DNA, generating positive supercoiling, in the presence of topoisomerases (Kimura and Hirano, 1997, Bazett-Jones et al., 2002). Recently, overwinding (leading to positive supercoiling) on yeast minichromosomes passing through mitosis was reported and was indeed shown to be dependent on condensin (Baxter et al., 2011). Importantly, Top2 showed a bias toward decatenation on catenated plasmids that were positively supercoiled but not negatively supercoiled (Baxter et al., 2011). These findings experimentally support the possibility that chromosome condensation facilitates Top2’s role in removing SCIs during mitosis (Koshland and Strunnikov, 1996).

The present study was designed to investigate why SCIs are maintained on chromosomes until metaphase and to test whether Top2 has a bias toward decatenation in its strand-passage activity during the G_2_/M period of the yeast cell cycle. Our findings reveal that SCIs are formed during G_2_/M by Top2-dependent catenation, and also show that the physical proximity of sister chromatids facilitates SCI formation. Finally, we present data supporting the proposed role of condensin in promoting directionality in Top2’s strand-passage activity to favor decatenation at the anaphase onset.

## Results

### SCIs Are Maintained in Prolonged Mitotic Arrests

**Figure 1:**
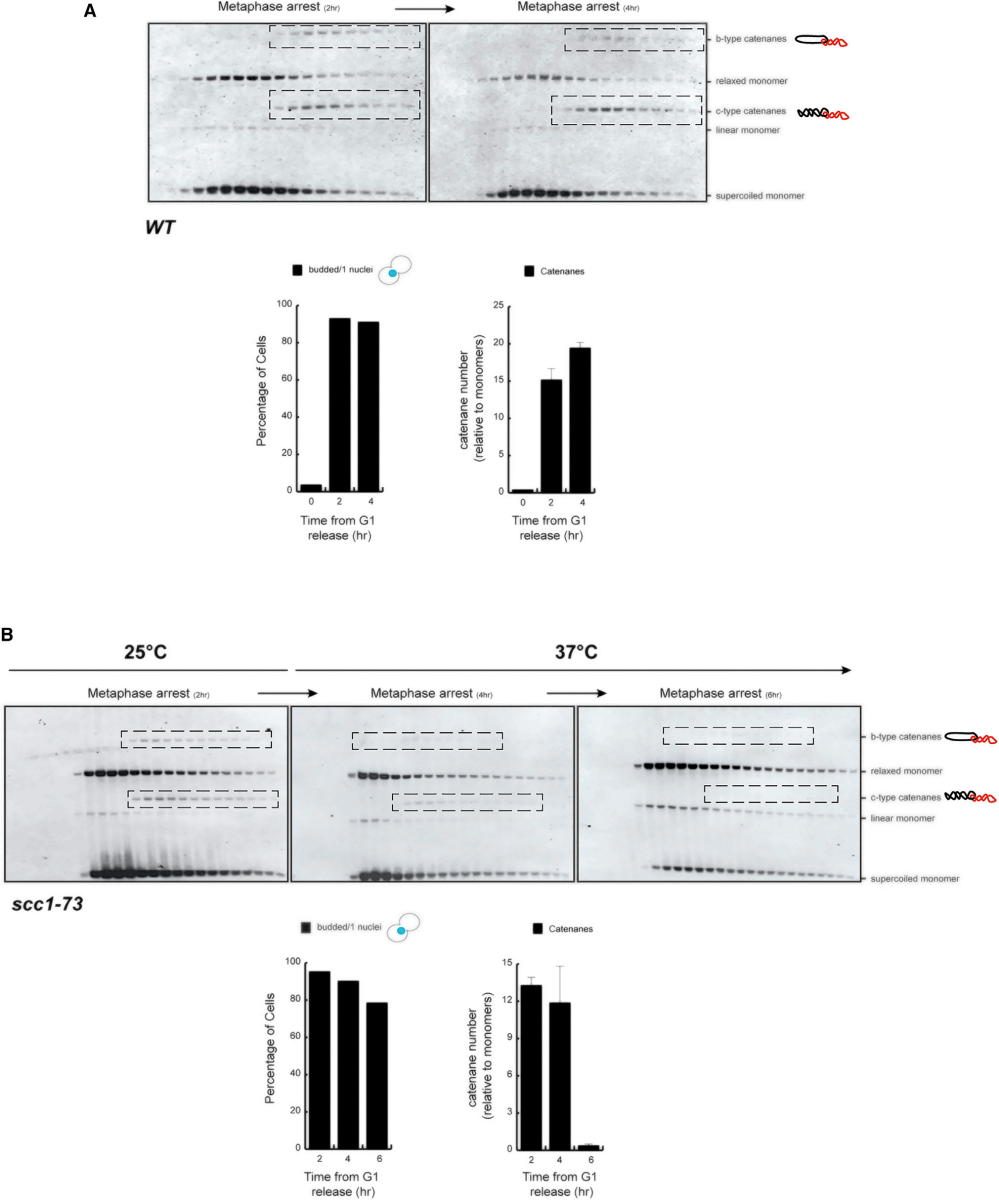
Sister Chromatid Intertwines Are Maintained in Prolonged Mitotic Arrests and Depend on Cohesin (A) The wild-type strain bearing a 10 kb circular minichromosome was synchronized in G_1_ and released into the cell cycle in the presence of nocodazole at 30°C. A second dose of nocodazole was added after 180 min. Samples were taken for analysis at 120 min and 240 min after release. Cell extracts were separated by differential sedimentation, heat denatured in 1% SDS, and analyzed by Southern blotting to reveal plasmid species. Plasmid species included monomeric forms (OCm, relaxed monomer; Lm, linear monomer; and CCCm, supercoiled monomer) and dimeric forms, including different sister chromatid intertwine (SCI) species (Catc-type catenanes and Catb-type catenanes). SCIs are indicated in the blots (dashed boxes). The mitotic arrest was stable during the experiment. SCI levels were maintained during the extended mitotic block. SCIs (including c- and b-type catenanes) were quantified using ImageJ software (bottom right-hand graph; showing the mean ± SD from three independent experiments). (B) The *scc1-73* strain bearing the 10 kb circular minichromosome was synchronized in G_1_ and released into the cell cycle in the presence of nocodazole at 25°C; upon metaphase arrest, the culture was shifted to 37°C to inactivate *scc1-73*. A second dose of nocodazole was added after 180 min. Samples were collected 0, 120, and 240 min after the temperature shift. Samples were analyzed as in (A). *scc1-73* inactivation in metaphase-arrested cells led to the progressive disappearance of SCI species. SCIs were quantified as in (A) (bottom right-hand graph; showing the mean ± SD from three independent experiments).

SCIs are thought to be formed during DNA replication (Postow et al., 2001) and removed progressively by Top2 during G_2_ and early mitosis (Stuchinskaya et al., 2009). Analysis of yeast centromeric plasmids, however, has revealed that in cells arrested in metaphase in the absence of spindles (i.e., arrests mediated by the microtubule poison nocodazole), SCIs persist (Farcas et al., 2011, Koshland and Hartwell, 1987). First, we used differential sedimentation velocity and gel electrophoresis of large yeast centromeric plasmids (Farcas et al., 2011) to test whether catenated dimers are preserved in a prolonged mitotic arrest in the absence of spindle microtubules (nocodazole arrests). Cell extracts were sedimented in 10%–45% sucrose gradients, and the plasmid-containing fractions were denatured by heating to 65°C in 1% SDS before being electrophoresed through 0.5% agarose gels. Southern blotting with plasmid-specific probes revealed both catenated sister DNA dimers and monomeric DNAs (Figure 1A). The electrophoretic mobility of monomers and dimers was similar to that observed in previous reports using this technique (Farcas et al., 2011). The identities of the electrophoretic forms were confirmed using digestion analysis (Figure S1). To evaluate changes in SCI levels over time, cells were blocked in metaphase with nocodazole and either immediately processed for analysis or kept arrested for extended times. Sucrose gradient fractions were analyzed, and the quantity of dimers (Catc/bs) was calculated relative to either the total amount of monomers (supercoiled/relaxed) (Figure 1A) or total DNA (Figure S2). The results revealed an increase in SCI quantities in samples that had been blocked with nocodazole for extended times (Figure 1A). Therefore, the level of catenated dimers did not diminish over time, as was expected from the assumption that Top2 progressively removes SCIs.

### Physical Proximity between Replicated Chromatids Is Required for the Maintenance of SCIs

Previous studies have demonstrated that inactivation of cohesin during interphase prevents the presence of intertwining on yeast plasmids in nocodazole-arrested cells during metaphase (Farcas et al., 2011). This has led to the proposal that cohesin protects SCIs from Top2-mediated decatenation (Farcas et al., 2011). First, we measured the kinetics of SCIs in nocodazole-blocked cells upon cohesin inactivation. Following the temperature shift in cells which were already blocked in nocodazole, to inactivate *scc1-73*, we observed the disappearance of SCIs over a 4-hr period (Figure 1B). Therefore, as shown previously for interphase (Farcas et al., 2011), inactivation of cohesin in metaphase-arrested cells also causes SCI loss.

**Figure 2:**
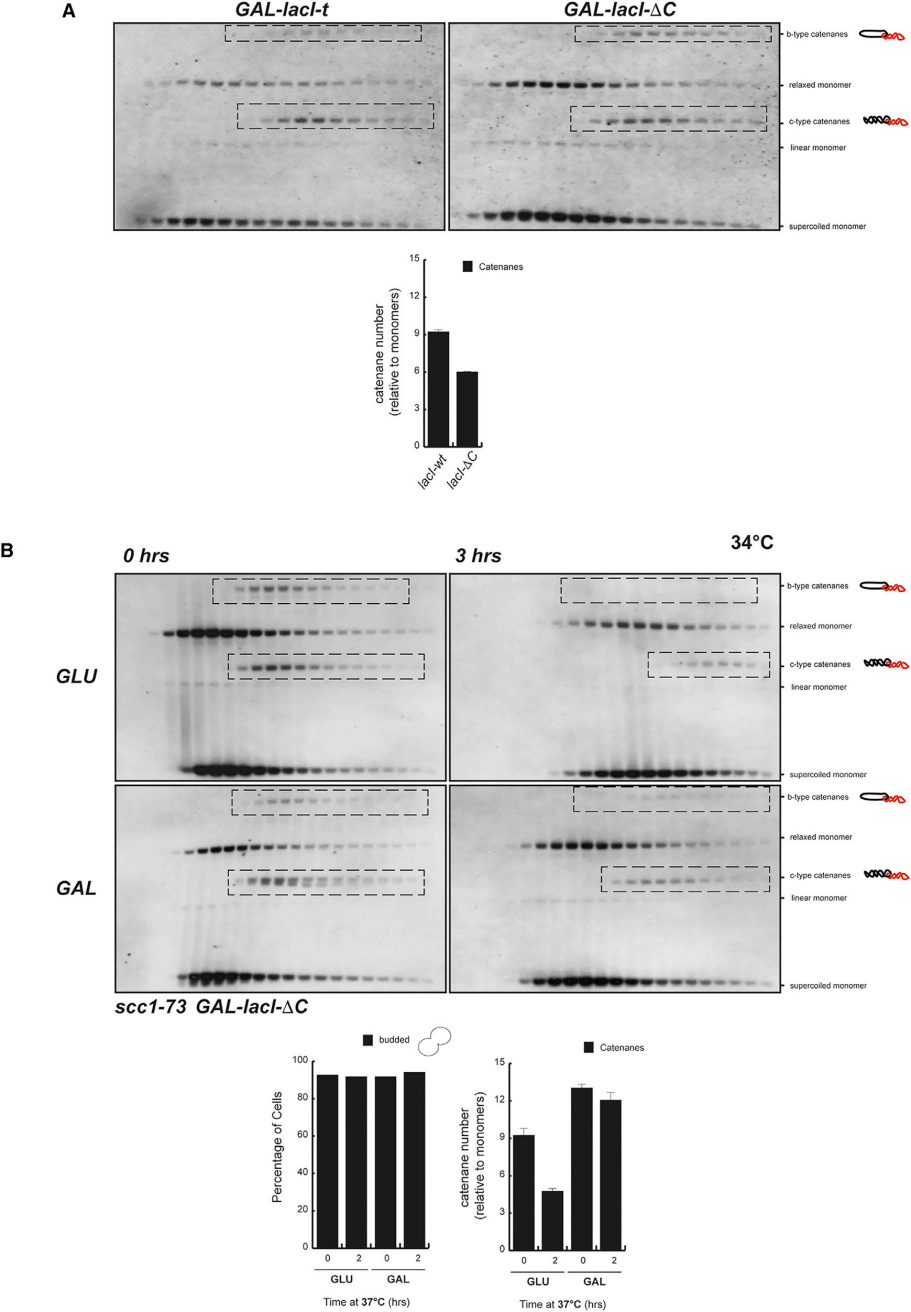
Physical Proximity between Sister Chromatids Maintains SCIs Post-replication (A) Strains bearing a 10 kb circular minichromosome containing short *lacO* arrays and expressing a wild-type *lacI* (*lacI-t*; able to bind two operators simultaneously) or a *lacI* with a C-terminal deletion (*lacI-ΔC*; able to bind a single operator) under the galactose promoter were synchronized in G_1_ and released into the cell cycle in the presence of galactose and nocodazole at 30°C. Cell extracts were separated by differential sedimentation, heat denatured in 1% SDS, and analyzed by Southern blotting to reveal plasmid species. Plasmid species included monomeric forms (OCm, Lm, and CCCm) and dimeric forms, including different SCI species (Catc-type catenanes and Catb-type catenanes). SCIs are indicated in the blots (dashed boxes). SCIs (including c- and b-type catenanes) were quantified relative to monomeric species using ImageJ software (right-hand graph; showing the mean ± SD from three independent experiments). Cells expressing *lacI-t* exhibited a small increase in SCI levels. (B) The *scc1-73* strain bearing the 10 kb circular minichromosome containing the *lacO* array and the *lacI-t* construct under the galactose promoter was grown in raffinose media and synchronized in G_1_. The culture was divided in two. One half was released in galactose media (expressing *lacI-t*) while the other half was released in glucose media (repressing *lacI-t*). Both cultures were released from the G_1_ arrest in the presence of nocodazole at 25°C, and upon metaphase arrest, the cultures were shifted to 34°C to inactivate *scc1-73*. Samples were analyzed at the metaphase block at 25°C and 3 hr after temperature shift. Analysis was done as in (A). *scc1-73* inactivation led to reduced SCI levels in the absence of *lacI-t* expression, but not in its presence. SCIs were quantified as in (A) (bottom right-hand graph; showing the mean ± SD from three independent experiments).

Next, we sought to investigate whether the maintenance of SCIs requires either physical proximity between sister chromatids or a function of the cohesin complex other than keeping chromatids together (i.e., direct protection of catenanes from Top2-mediated decatenation). To address this, we decided to maintain physical proximity between sister plasmids in the absence of functional cohesin. Our minichromosomes carried a short stretch of lactose operators, and we expressed two forms of lactose repressors: one that binds a single operator (*lacI-ΔC*), and another capable of binding two operators simultaneously (*lacI-t*) (Straight et al., 1996). This approach has been previously used to hold sister chromatids in the absence of cohesin (Straight et al., 1996) (Figure S3A). We compared SCIs on *lacO*-bearing plasmids in cells expressing either *lacI-t* or *lacI-ΔC*. Both *lacI* forms were expressed using the galactose-inducible promoter. The level of SCIs was greater in cells expressing *lacI-t* (Figure 2A), suggesting that increased closeness between chromatids might promote SCIs. Next, we inactivated cohesin using the *scc1-73* allele in cells expressing *lacI-t* (Figure 2B). *scc1-73* cells carrying the *lacI-t* system were arrested in metaphase, and *lacI-t* was expressed as *scc1-73* was inactivated (Figure 2B).

Note that inactivation of *scc1-73* was achieved by raising the temperature to 34°C instead of 37°C because we found that expression of *lacI-t* from the galactose promoter was not efficient at 37°C (data not shown); however, cohesion loss in *scc1-73* cells was confirmed at 34°C (Figure S3B). As before, inactivation of *scc1-73* led to loss of SCIs (Figure 2B, GLU); however, *lacI-t* expression reduced SCI loss significantly (Figure 2B, GAL). From these results, we conclude that physical proximity between chromatids is a key parameter required for the maintenance of SCIs in cells arrested in metaphase by nocodazole treatment. Therefore, the loss of SCIs upon cohesin inactivation is a consequence of the separation between sister DNAs, rather than a loss of inhibition by cohesin on Top2 function.

### Increased Levels of Top2 Promote Formation of SCIs in Metaphase

**Figure 3:**
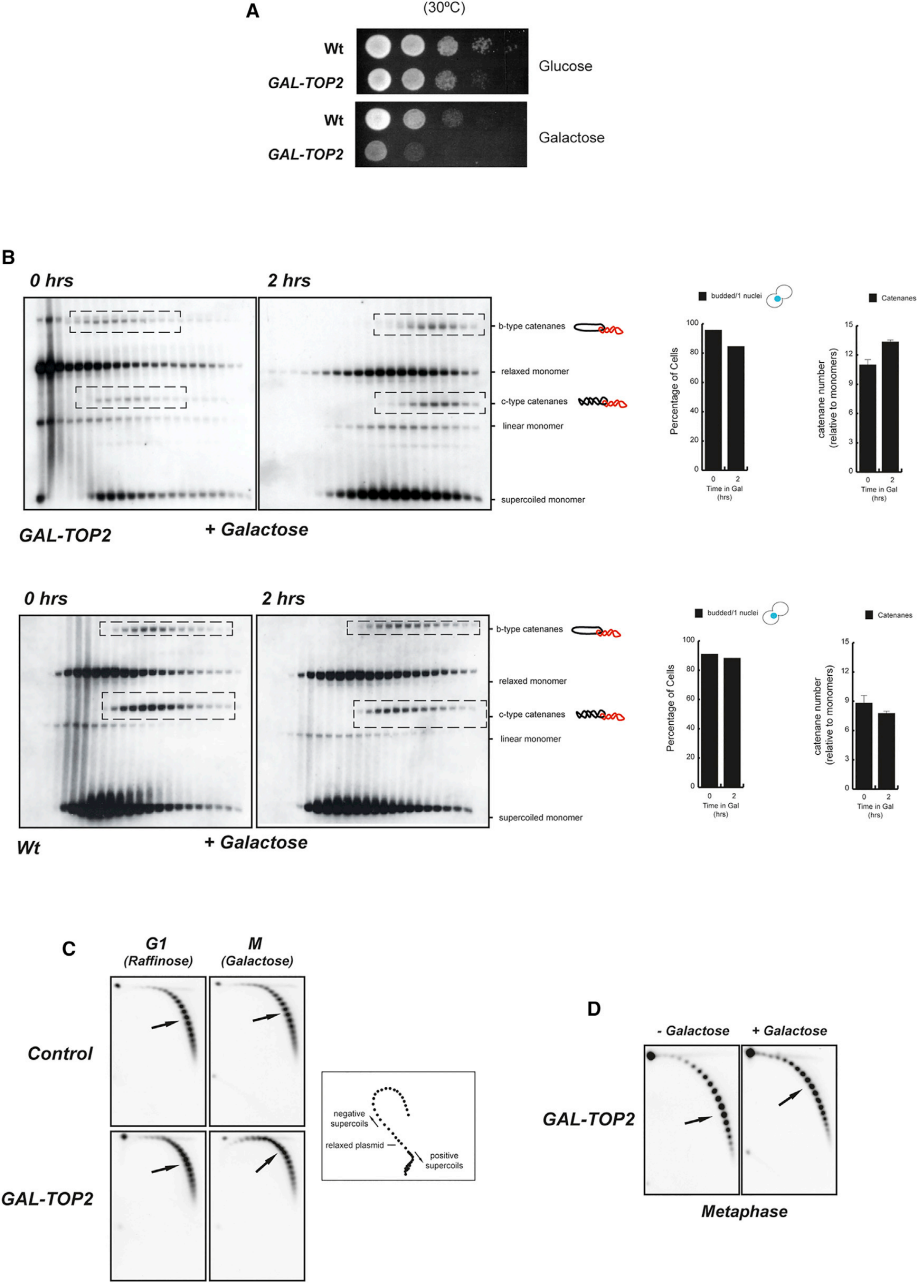
Increased Levels of Top2 Promote SCI Formation (A) A strain carrying a chromosomal insertion of *GAL-TOP2* in the *ura3* locus and a wild-type strain were grown and plated on media containing glucose (where *GAL-TOP2* is not expressed) or galactose (where *GAL-TOP2* is expressed) at 30°C. Images were taken after 3 days. Expression of *TOP2* from the *GAL1-10* promoter impaired cellular growth. (B) Wild-type and *GAL-TOP2* strains bearing the 10 kb circular minichromosome were synchronized in G_1_ in media containing raffinose and released into the cell cycle in the presence of nocodazole at 30°C. Following metaphase arrest, cells were transferred to new media containing galactose and nocodazole to activate expression of *TOP2* from the *GAL1-10* promoter. Samples were taken for analysis just before and 120 min after transfer to galactose. Cell extracts were separated by differential sedimentation, heat denatured in 1% SDS, and analyzed by Southern blotting to reveal plasmid species. Plasmid species included monomeric (OCm, Lm, and CCCm) and dimeric forms, including different SCI species (Catc-type catenanes and Catb-type catenanes). Running position for SCIs in the blots are indicated (dashed boxes). SCIs (including c- and b-type catenanes) were quantified using ImageJ software (right-hand graphs; showing the mean ± SD from three independent experiments). SCIs increased upon activation of *GAL-TOP2*. (C) *GAL-TOP2* and control strain bearing a circular minichromosome (pRS316) were blocked in G_1_ in media containing raffinose and released into the cell cycle in the presence of galactose and nocodazole at 30°C. DNA was purified from cells in the G_1_ arrest (G_1_− Raffinose) and at the metaphase block (M−Galactose) and analyzed by two-dimensional gel chloroquine analysis to assess the supercoiling status of monomer plasmids in the cells. A small distribution change toward a less negatively supercoiled population was observed upon *TOP2* expression (bottom panels). A cartoon representation of how the plasmid distribution relates to supercoiling status is shown. (D) *GAL-TOP2* bearing a circular minichromosome (pRS316) was blocked in G_1_ in media containing raffinose and released to nocodazole in raffinose media at 30°C. Upon metaphase arrest, half of the culture was maintained in raffinose while galactose was added to the other half to induce *TOP2* expression. Samples were taken 120 min after the galactose addition. DNA was purified and analyzed as in (C). A small distribution change toward a less negatively supercoiled population was observed upon *TOP2* expression.

Our results show that the physical proximity of chromatids is important for the maintenance of SCI levels in nocodazole arrests (Figure 2B). This suggests that, rather than blocking Top2-dependent decatenation, cohesin rings might facilitate the reverse reaction (i.e., catalysis of sister DNA catenation) by bringing about closeness of sister DNAs. Early in vitro studies using *X. laevis* egg extracts showed that increased levels of Top2 led to higher catenation of DNA substrates (Baldi et al., 1980). Therefore, we reasoned that increasing the amount of cellular Top2 might potentially alter SCI levels in chromosomes. It is noteworthy that overexpression of Top2 from the *GAL1-10* promoter is detrimental to cell growth (Figure 3A) (Vosberg, 1985, Worland and Wang, 1989). We arrested wild-type cells carrying an additional copy of Top2 under the *GAL1-10* promoter in metaphase using nocodazole, and we induced Top2 expression with galactose. Analysis of sucrose gradient fractions showed that SCI levels increased after Top2 expression (Figure 3B). This increase was not observed in cells carrying a control plasmid (Figure 3B). Therefore, we conclude that the quantity of Top2 protein inside cells affects the levels of SCIs in the minichromosomes.

We noticed that Top2 overexpression caused shifting of plasmid distributions on the gradients (Figure 3B), including monomer and dimer forms. It is possible that, in addition to altering SCI levels, additional Top2 leads to changes in the supercoiling status of plasmids, thus explaining the altered distribution of plasmids on the gradients. To investigate this, we compared the supercoiling of monomer plasmids in the presence or absence of Top2 overexpression (Figure 3C). Cells were released from G_1_ into metaphase in the presence or absence of Top2 overexpression. DNA was purified at both the G_1_ and metaphase arrests and then analyzed in 2D chloroquine gels to reveal the supercoiling distribution of the plasmids. No change in the distribution of topoisomers was observed when cells did not overexpress Top2 (Figure 3C, Control). In contrast, a small deviation toward a less negative topoisomer distribution was observed in cells expressing additional Top2 (Figure 3C). Similar results were observed when Top2 expression was induced in cells arrested in metaphase (Figure 3D). We conclude that changes in supercoiling contribute to the shifting of plasmid distributions on the gradients upon Top2 expression.

### Increasing Levels of Cellular Top2 Negatively Affect Sister Chromatid Segregation

**Figure 4:**
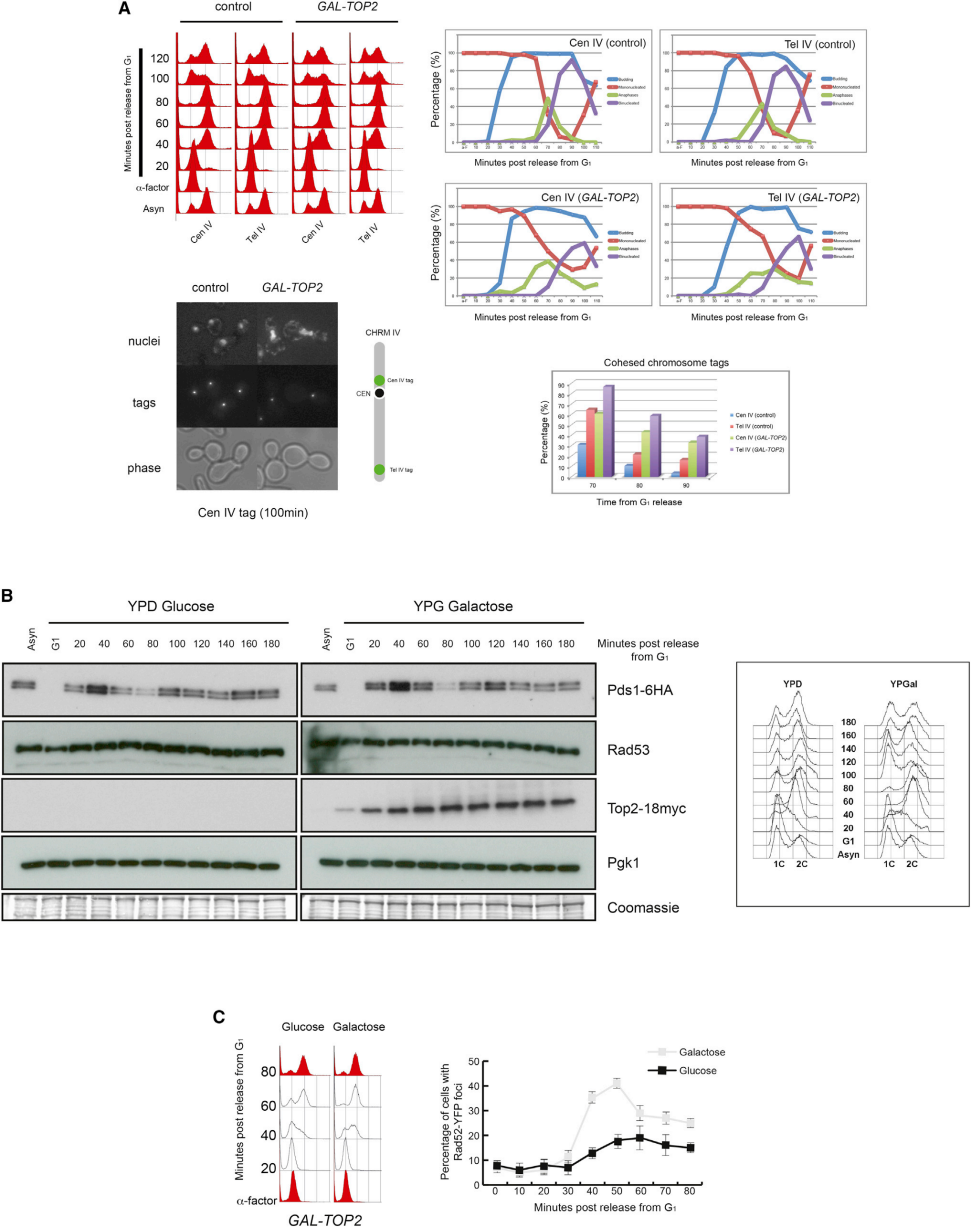
Increased Levels of Cellular Top2 Negatively Affect Sister Chromatid Separation (A) Wild-type and *GAL-TOP2* strains, carrying chromosomal tag (*tetO*) insertions in the vicinity of the centromere (Cen IV) or right telomere (Tel IV) of chromosome IV, were synchronized in G_1_ in media containing raffinose and released into the cell cycle in the presence of galactose at 30°C. Cytological analysis involved imaging 200 cells per time point in each replicate experiment. Percentage of mononucleated cells, binucleated cells, and anaphases (stretched DNA mass across bud-neck), together with the budding index, is shown for each strain. Sister chromatid separation dynamics in the strains was measured by scoring cohesed chromosomal tags at time points between 70 and 90 min (bottom graph). FACS profile for each strain is shown. Representative micrographs for wild-type and *GAL-TOP2* strains carrying centromeric tags are shown. Diagrammatic representation of the position of the chromosomal tags within chromosome IV is indicated. (B) A strain carrying *GAL-TOP2-18myc Pds1-6HA* was synchronized in G_1_ in media containing raffinose and released into the cell cycle in the presence of glucose or galactose at 30°C. Samples were taken for analysis at the times indicated. Top2 induction, Pds1 degradation, and Rad53 phophorylation during the time courses were followed by western blot. FACS profile is shown. No changes in Rad53 phosphorylation or Pds1 degradation timing were observed in cells overexpressing *TOP2*. (C) A *GAL-TOP2* strain bearing *RAD52-YFP* constructs was synchronized in G_1_ in media containing raffinose and released into the cell cycle in the presence of glucose or galactose at 30°C. Samples were taken for analysis every 10 min as the cultures proceeded through S phase and analyzed microscopically for Rad52 foci formation and by FACS. Cytological analysis involved imaging 200 cells per time point in each replicate experiment.

The changes observed in supercoiling and the increase in SCIs upon Top2 overexpression (Figures 3B–3D) prompted us to investigate effects on chromosome segregation. To test this, we expressed Top2 from the *GAL1-10* promoter in G_1_-arrested cells before releasing them into the cell cycle, and we visualized the timing of sister chromatid segregation. We employed chromosome tags in the vicinity of the centromere and telomere of chromosome IV. Overexpression of Top2 caused delays in separation of both centromeric and telomeric regions (Figure 4A). Stretched anaphase nuclei accumulated and persisted in cells overexpressing Top2 for longer times than in control cells (Figure 4A), demonstrating that Top2 expression adversely affected sister chromatid disjunction. However, cells eventually segregate nuclear masses normally. The chromosome segregation delay was not caused by delayed cell-cycle progression, as the kinetics of Pds1 (Securin) degradation was similar in Top2 overexpression and control samples (Figure 4B). Furthermore, we did not observe activation of the DNA damage checkpoint kinase Rad53 in cells expressing Top2 (Figure 4B), which shows that a DNA damage checkpoint response is not triggered when Top2 levels are increased. Nevertheless, a transient increase in Rad52 foci was observed during S phase in cells overexpressing Top2 (Figure 4C), suggesting higher levels of ongoing DNA repair in these cells. These results raise the possibility that increased levels of Top2, despite not triggering a checkpoint response, might have detrimental effects on long-term genome stability.

### Condensin Is Required for SCI Resolution

SCIs are maintained on cohesed metaphase minichromosomes because of their physical proximity (Figure 2B). Condensin complexes promote mitotic chromosome condensation and are required for sister chromatid resolution during segregation in anaphase (Hirano, 2012). Although the inactivation of condensin during interphase does not affect SCI levels on metaphase minichromosomes arrested with nocodazole (Farcas et al., 2011), it prevents the full removal of SCIs by telophase (Charbin et al., 2014). Surprisingly, in cells arrested in metaphase by the spindle poison nocodazole, SCIs are maintained on minichromosomes, while in cells arrested in metaphase through depletion of the anaphase-promoting complex (APC) activator Cdc20, SCIs are resolved (Charbin et al., 2014, Farcas et al., 2011). The difference between these two metaphase arrests lies in the presence of spindles in the Cdc20-mediated block. Incidentally, condensin binds to pericentromeric regions of replicated chromatids when nocodazole is present in Cdc20-mediated blocks, and spreads to chromosome arms as bipolar spindles are allowed to form (upon nocodazole removal in Cdc20-depleted conditions) (Leonard et al., 2015). Importantly, condensin relocalization temporally correlates with its function in promoting overwinding (positive supercoiling) of DNA (Baxter et al., 2011, Leonard et al., 2015), which requires Polo- and Aurora-kinase-dependent phosphorylation (Leonard et al., 2015, St-Pierre et al., 2009).

**Figure 5:**
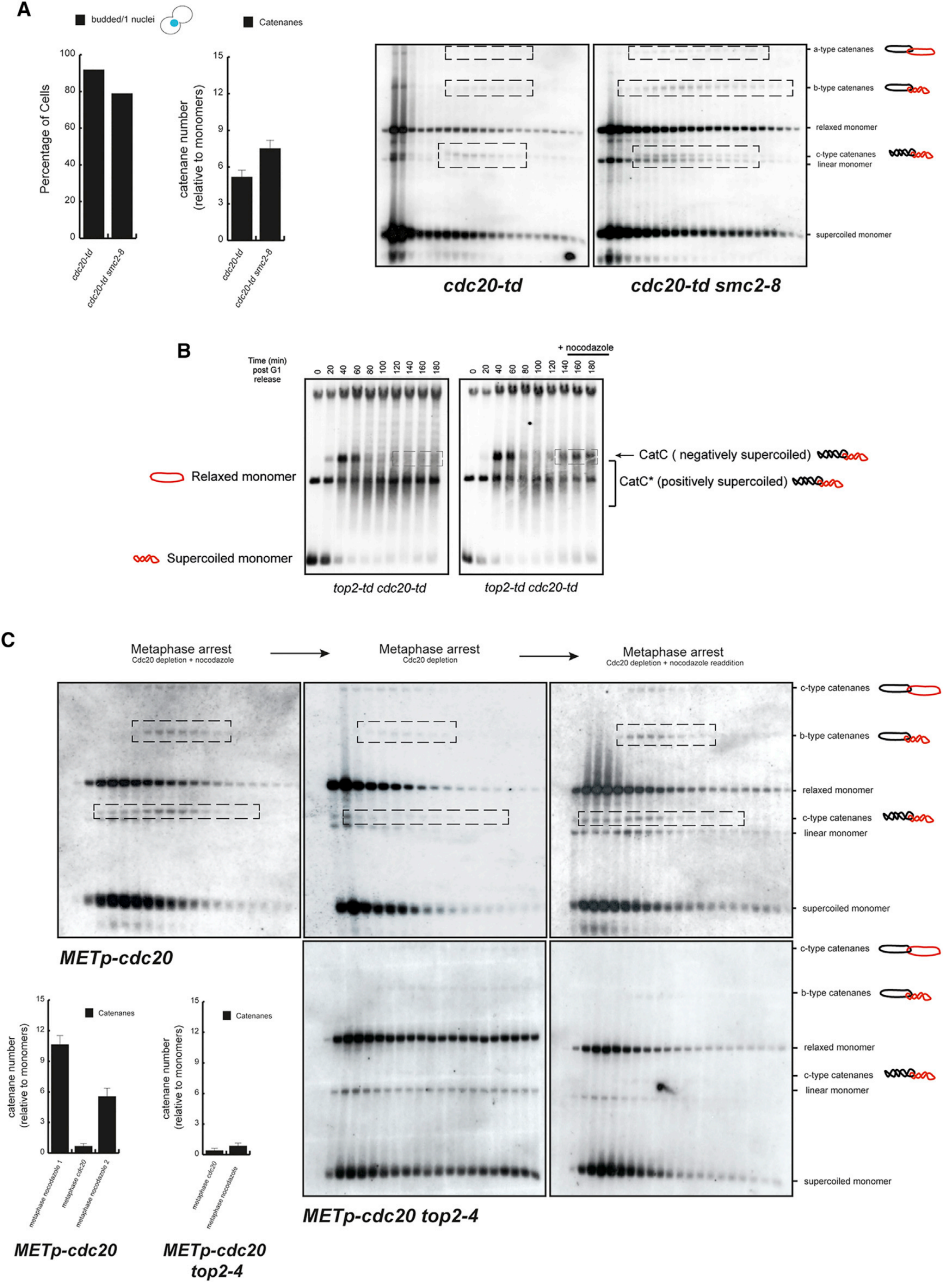
Reversing Condensin-Dependent Overwinding Allows Top2-Dependent SCI Formation in Metaphase Cells (A) *cdc20-td* and *cdc20-td smc2-8* strains bearing the 10 kb circular minichromosome were synchronized in G_1_ and released into the cell cycle under conditions of Cdc20 depletion at 37°C. Samples were taken at the metaphase block. Cell extracts were separated by differential sedimentation, heat denatured in 1% SDS, and analyzed by Southern blotting to reveal plasmid species. Plasmid species included monomeric forms (OCm, Lm, and CCCm) and dimeric forms, including different SCI species (Cata-, Catb-, and Catc-type catenanes). Running position for SCIs in the blots is indicated (dashed boxes). The mitotic arrest was stable during the experiment. SCIs were increased in the *cdc20-td smc2-8* strain. SCIs (including a-, b-, and c-type catenanes) were quantified using ImageJ software (bottom right-hand graph; showing the mean ± SD from three independent experiments). (B) A *cdc20-td top2-td* strain bearing the centromeric plasmid pRS316 was synchronized in G_1_, split in two, and released into the cell cycle under conditions of Cdc20 and Top2 depletion. Nocodazole was added to one of the samples after 120 min. Samples were collected every 20 min and DNA was resolved in agarose gels and probed for the circular minichromosome. Electrophoretic mobilities of monomers (OCm, Lm, and CCCm) are indicated. Dimeric forms for CatC-type catenanes (negatively supercoiled) and CatC^∗^-type catenanes (positively supercoiled) (Baxter et al., 2011) are also indicated. Addition of nocodazole led to the shift back from CatC^∗^ to CatC. (C) A *METp*-*CDC20* strain (Cdc20 under Methionine promoter) (top panel) bearing the 10 kb circular minichromosome was synchronized in G_1_ and released into the cell cycle in the presence of nocodazole under Cdc20-depleting conditions (YPD + 5 mM methionine) at 36°C. Following metaphase arrest, cells were transferred to new media lacking nocodazole but maintaining the Cdc20 depletion (hence allowing formation of mitotic spindles) for 120 min before readdition of nocodazole. Samples were taken for analysis at the first nocodazole block (metaphase arrest − Cdc20 depletion + nocodazole), 120 min after nocodazole removal (metaphase arrest − Cdc20 depletion) and 120 min after nocodazole readdition (metaphase arrest − Cdc20 depletion + nocodazole readdition). A *METp*-*CDC20 top2-4* (bottom panel) bearing the 10 kb circular minichromosome was synchronized in G_1_ and released into the cell cycle under conditions of Cdc20 depletion (YPD + 5 mM methionine) at 25°C. Nocodazole was added to the culture, and the temperature was shifted to 36°C to inactivate Top2. Samples were taken at Cdc20 block and 120 min after nocodazole addition. Cell extracts were separated by differential sedimentation, heat denatured in 1% SDS, and analyzed by Southern blotting to reveal plasmid species. Plasmid species included monomeric (OCm, Lm, and CCCm) and dimeric forms, including different SCI species (Catc-type catenanes and Catb-type catenanes). Running position for SCIs in the blots is indicated (dashed boxes). The mitotic arrest was stable during the experiments. SCIs (including c- and b-type catenanes) were quantified using ImageJ software (bottom left-hand graph; showing the mean ± SD from three independent experiments). SCIs were reduced when nocodazole was removed, and reappeared upon its readdition in the presence of Top2, but not in its absence.

Since this function has been proposed to provide a bias for Top2 toward decatenation (Baxter et al., 2011), we considered the possibility that the hyperactivation of condensin supercoiling promotes SCI removal on cohesed chromatids despite their physical proximity. To test this, we investigated whether or not removal of SCIs occurs in metaphase arrests by Cdc20 depletion when condensin function is compromised. To this end, we used the conditional allele *smc2-8.* Analysis of sucrose gradient fractions confirmed that *cdc20-td smc2-8* cells have higher levels of SCIs than *cdc20-td* cells do (Figure 5A). This result shows a correlation between hyperactivation of condensin supercoiling and SCI resolution in metaphase blocks where physical proximity of chromatids due to cohesion is still present.

### Reversing Condensin-Dependent Overwinding Allows Top2-Dependent SCI Formation in Metaphase Cells

Analysis of the genome-wide distribution of yeast condensin has shown that the complex binds to centromere regions of replicated chromatids in G_2_/M arrests with nocodazole (Leonard et al., 2015), while in Cdc20-mediated blocks condensin spreads to chromosome arms (Leonard et al., 2015). However, the addition of nocodazole to Cdc20-mediated blocks restores the centromeric localization of condensin (Leonard et al., 2015).

Since condensin-dependent overwinding requires chromosomes to be attached to mitotic spindles (Baxter et al., 2011), we decided to test whether condensin overwinding is inhibited when nocodazole is added to Cdc20-mediated blocks. Overwinding can be detected as a change in the electrophoretic mobility of yeast minichromosomes in the absence of Top2 activity (Baxter et al., 2011, Leonard et al., 2015). The mobility shift is caused by a transition from negatively supercoiled (CatC) to positively supercoiled (CatC^∗^) catenanes (Baxter et al., 2011, Leonard et al., 2015). We released cells from G_1_ in the absence of Top2 and Cdc20 (Figure 5B, *top2-td* and *cdc20-td*) and analyzed the formation of CatC^∗^s (overwound, or positively supercoiled, dimers). We first detected CatCs (underwound, or negatively supercoiled, dimers) 40–60 min after release (Figure 5B). These CatCs transitioned to CatC^∗^s from 80 min onward as the cells reached metaphase arrests by Cdc20 depletion (Figure 5B). We performed two experiments in parallel, and added nocodazole to one of them 120 min after the G_1_ release (a time when the CatC-to-CatC^∗^ transition had taken place). CatCs reappeared when nocodazole was added back to Cdc20-blocked cells (Figure 5B). Therefore, overwinding can be inhibited in Cdc20-blocked cells by the addition of nocodazole.

Next, we reasoned that, if overwinding provides a bias toward decatenation for Top2 action, as predicted from our results (Figure 5A), then upon nocodazole inhibition of overwinding in Cdc20 blocks (Figure 5B), Top2 should lose its directionality toward decatenation; as a consequence, new SCIs should be reformed between minichromosomes. First we confirmed that, in Cdc20 blocks containing nocodazole, SCIs are resolved when nocodazole is removed from media (Figure 5C). Next, we added nocodazole back to Cdc20-blocked cells that had resolved SCIs (Figure 5C). As predicted, nocodazole addition to Cdc20 blocks led to the reappearance of new SCIs (Figure 5C), and this was dependent on Top2 (Figure 5C). Therefore, our data suggest that condensin-dependent overwinding provides a bias for Top2 action toward decatenation when cohesed chromosomes are attached to spindle microtubules, and that inhibiting overwinding reverts Top2 strand-passage activity to work bidirectionally, as it does during the post-replicative period of G_2_, a time of the cell cycle when Top2 action not only removes SCIs between sister chromatids, but also promotes them.

## Discussion

Pioneering studies on SV40 replication termination first linked the formation of SCIs to the DNA replication process (Sundin and Varshavsky, 1980). Based on the intimate connection between DNA replication and SCI formation, and the fact that these structures can keep sister chromatids together, SCIs were the first mechanism proposed to explain sister chromatid cohesion (Murray and Szostak, 1985). However, the realization that in Cdc20 mutants yeast minichromosomes are cohesed in the absence of SCIs (Koshland and Hartwell, 1987) ruled out SCIs as the major cohesion force and provided the intellectual framework for the discovery of cohesins as the protein-mediated bridges that hold sister chromatids (Guacci et al., 1997, Losada et al., 1998, Michaelis et al., 1997). This was followed by a detailed description of the cell-cycle regulation behind cohesin removal in anaphase (Nasmyth, 2001), which implied that cells regulate cohesin to hold sisters and resulted in reduced interest in SCIs. In addition, in vitro studies of Top2 demonstrated that this enzyme decatenates plasmids below the expected thermodynamic equilibrium (Rybenkov et al., 1997), an observation that led to the proposal that Top2 has directionality toward decatenation. Therefore, SCIs were predicted to form exclusively during DNA replication and to be rapidly removed by Top2 during G_2_, with only a minor population of SCIs remaining until mitosis. Here, we have uncovered several unexpected facts about SCIs. First, we have shown that SCI formation is not limited to the process of DNA replication or the S phase period of the cell cycle. Second, we have demonstrated that Top2 does not have an intrinsic directionality toward decatenation. Third, we identified physical proximity between chromatids and Top2 cellular amounts as key parameters behind the replication-independent catenation of chromatids. And finally, we have shown that condensin overwinding, which requires mitotic spindles, correlates with the initiation of Top2 decatenation on cohesed chromosomes despite their physical proximity, suggesting that directionality toward decatenation relies on condensin overwinding. Moreover, our results fully explain why SCIs are present in metaphase arrests with nocodazole but absent in metaphase blocks mediated by Cdc20 depletion, in which spindles are present.

The view that SCIs are rapidly dissolved by Top2 following DNA replication has been recently called into question by the observation that a significant percentage of SCIs persist in large yeast minichromosomes when cells are arrested in metaphase with the microtubule poison nocodazole (Farcas et al., 2011). In addition, studies on mammalian chromosomes using Top2 inhibitors have demonstrated that a significant number of catenations persist until mitosis, leading to the proposal that these catenations are removed during the process of chromosome individualization (Giménez-Abián et al., 1995, Losada et al., 2002). The persistence of SCIs after DNA replication in yeast minichromosomes was shown to depend on cohesin (Farcas et al., 2011). We considered two potential mechanisms to explain the requirement of cohesin for SCI maintenance: an “SCI protection” feature by cohesin in which the complex masks SCIs from Top2’s resolution, or a “stimulation of concatenation” scenario brought about by the proximity of sister DNAs at cohesin sites. The fact that SCIs are maintained in minichromosomes held together by tetramerized *lacI* (Figure 2B) and the observed de novo formation of SCIs when condensin-dependent overwinding is inactivated in mitosis (Figure 5C) demonstrates that cohesin’s role in maintaining SCIs is indirect, and simply a consequence of the fact that cohesion promotes physical proximity between chromatids. It is noteworthy that previous biochemical analysis of purified cohesin from HeLa cells showed that the complex stimulates intermolecular catenation of circular DNA molecules in the presence of Top2 (Losada and Hirano, 2001). Our findings extend these observations and show that in vivo cohesin sites represent regions where SCI links are formed by Top2 between sister chromatids.

It is important to recognize that the formation of SCIs at cohesin sites does not represent an active, or cohesin-independent, mechanism to maintain sister chromatid cohesion, since SCIs depend on cohesin presence during G_2_. Several studies have proposed the existence of multiple mechanisms, including SCIs, to ensure cohesion along chromosomes (Díaz-Martínez et al., 2008). It is evident that at any given time the presence of SCIs provides additional physical links between sister DNA molecules; however, the ongoing formation and resolution of SCIs by Top2 at cohesin sites would result in the rapid disappearance of SCIs without cohesin’s action in maintaining physical proximity. Consequently, SCIs would not be sufficient to maintain any form of sustainable cohesion between chromatids for extended periods of time. Therefore, in our view, SCIs represent a byproduct of cohesin function and not an active mechanism providing cohesion between chromatids.

Previous arguments against a direct role for SCIs in cohesion often invoked the fact that SCI removal was not thought to be cell-cycle regulated, unlike cohesin cleavage by separase. Contrary to this view, our results show that mechanisms exist to promote removal of SCIs from cohesed chromosomes at the anaphase onset. SCIs are present in minichromosomes arrested in metaphase using the spindle poison nocodazole, but they are resolved when mitotic spindles reform (Figure 5C). This demonstrates that removal of SCIs occurs after bipolar attachment of the mitotic spindles as the cells initiate anaphase. Indeed, we show that condensin-dependent overwinding, which is activated by spindle attachments (Baxter et al., 2011, Leonard et al., 2015), correlates with the removal of SCIs, suggesting a regulated mechanism for the clearance of SCIs from chromosomes at this stage. Pioneering studies with frog extracts showed that condensin overwinds DNA, generating positive supercoiling in the presence of topoisomerases (Kimura and Hirano, 1997). We recently demonstrated that yeast condensin promotes positive supercoiling of minichromosomes when spindle microtubules attach to kinetochores (Baxter et al., 2011, Leonard et al., 2015). To stabilize, and hence detect, the positive supercoiling status of monomer plasmids, Top2 inactivation was required (Baxter et al., 2011); this demonstrates that positively supercoiled plasmids are a strong substrate for Top2 relaxation activity. Importantly, human Top2 showed a bias toward decatenation when catenated plasmids were positively supercoiled, but not when they were negatively supercoiled (Baxter et al., 2011), suggesting that condensin-dependent overwinding promotes decatenation by Top2 through alteration of substrate topology (Baxter and Aragón, 2012). Here, we have been able to confirm that condensin overwinding on cohesed chromosomes leads to SCI removal, and that inhibition of this activity results in new SCI formation in metaphase (Figure 5C). Therefore, we propose that condensin overwinding upon bipolar attachment of sister chromatids to spindles represents a cell-cycle-regulated mechanism for SCI clearance in cohesed mitotic chromosomes.

During our investigation we observed that increasing the amounts of Top2 in cells caused the accumulation of SCIs and altered the supercoiling status of minichromosomes; this suggests that enzyme levels are critical when considering Top2 activities on chromatin. The cellular amount of Top2 is clinically relevant because patients with amplification of Top2 respond favorably to anthracycline-based chemotherapy (O’Malley et al., 2011). Indeed, it has been known for some time that increased levels of Top2 in mammalian cells lead to hypersensitivity to Top2-targeting drugs, whereas reduced levels cause resistance to these poisons (Nitiss and Beck, 1996). Our findings demonstrating that cellular levels of Top2 correlate with Top2 activity are consistent with these observations, since higher amounts of drug-induced DNA lesions can be expected with increased cellular levels, and hence activity, of Top2. The importance of cellular mechanisms maintaining Top2 homeostasis has been underestimated; our data suggest that either too little or too much Top2 in cells is likely to generate genome instability and chromosome loss over generations. It follows that strategies to modulate cellular Top2 levels might present a novel therapeutic opportunity, because Top2 poisons are widely used in human cancer treatment.

**Figure 6:**
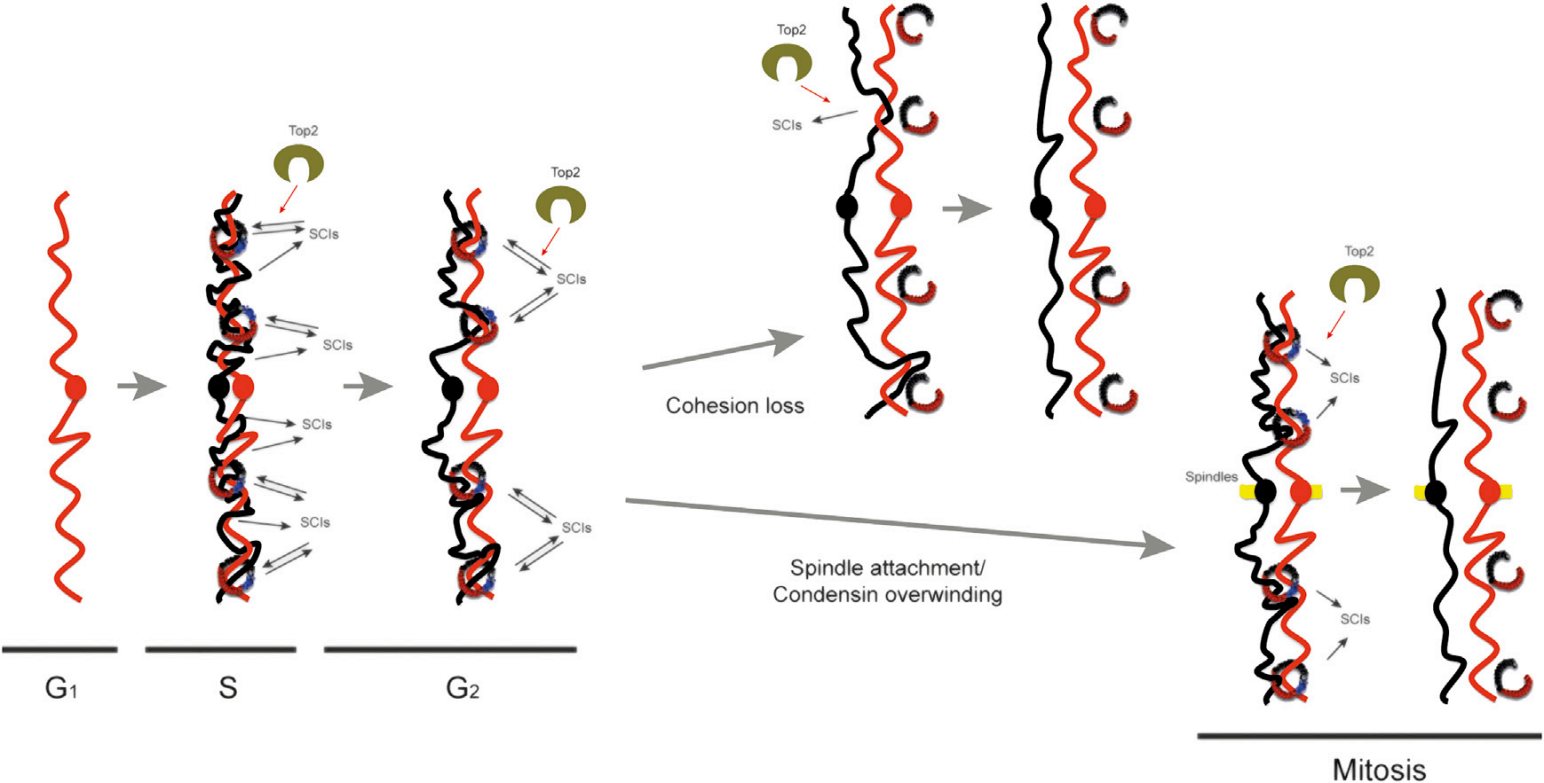
Diagrammatic Model for SCI Formation and Resolution during the Cell Cycle During DNA replication, pre-catenanes are formed as fork swiveling occurs, leading to the formation of SCIs upon replication completion. During G_2_, Top2’s bidirectional activity acts on SCIs by removing them, but also by catalyzing their formation de novo at cohesin sites, where the physical proximity between chromatids offers this opportunity. Therefore, cohesin sites represent regions of the genome where SCI levels are at equilibrium, i.e., where Top2’s SCI removal and catalysis co-exist. Catalysis of SCI formation at these regions is likely to be a consequence of the presence of intermolecular crossovers between sister chromatids at or adjacent to cohesin sites. If premature cohesion loss is induced by inactivation of cohesin, a reduction in SCI formation would ensue (because of the physical separation of chromatids); consequently, the global levels of SCIs would diminish. Upon bipolar spindle attachment, condensin action (promoting overwinding and chromosome compaction) enhances Top2’s monodirectional activity toward SCI resolution, potentially by generating distinct chromatid identities and reducing the probability of intermolecular crossovers between sister chromatids, so that when SCI removal occurs SCI catalysis is not favorable. Following condensin activation and SCI removal, proteolytic cleavage of cohesin’s subunit Mcd1 triggers anaphase chromosome segregation.

Our findings provide a new view for the mechanisms behind SCI formation and resolution (Figure 6). We propose that, during DNA replication, the bulk of SCIs are produced as a consequence of fork swiveling, particularly during replication completion. Following replication, the proximity of sister DNAs brought about by their pairing at cohesin sites facilitates the formation of SCIs between sister chromatids by Top2 (Figure 6). Importantly, we predict that Top2 activity at sites of physical proximity, cohesin sites, is bidirectional: that is, both formation and removal of SCIs occur at these sites (Figure 6), leading to a situation where the equilibrium results in a probability that the site will contain a given number of SCIs at any particular moment in time. If premature cohesion loss occurs, as is the case in cohesin mutants, a reduction in SCI formation would ensue because of the increased physical separation of chromatids; consequently, the global levels of SCIs would diminish. When cohesed sister chromatids become bioriented on the mitotic spindle, condensin-mediated overwinding alters DNA topology, in ways we do not yet understand, so that Top2 gains a bias toward decatenation and SCIs are removed in cohesed chromatids despite their physical proximity (Figure 6), leaving cohesin cleavage by separase as the last cellular task needed to separate the two daughter genomes.

## Experimental Procedures

### Media and Cell-Cycle Synchronizations

Yeast strains bearing the 10 kb plasmid were grown in synthetic media lacking tryptophan and supplemented with 2% glucose (−Trp+D) at 25°C. Exponentially growing cultures were subsequently diluted to OD_600_ = 0.25 in YEP (2% dextrose) media and allowed to grow to attain OD_600_ = 0.6–0.8 at 25°C. Cells were arrested at metaphase in the presence of 15 μg/mL nocodazole for 3 hr. Strains bearing the temperature-sensitive degron *cdc20-td* or *top2-td* with or without the temperature-sensitive allele *smc2-8* or *top2-4* were grown overnight at 25°C in minimal media lacking tryptophan −TRP (2% dextrose). Exponentially growing cultures were diluted to OD_600_ = 0.25 in YEP (2% raffinose) and allowed to grow for 8 hr before being diluted to OD_600_ = 0.05 in YEP (2% raffinose) and allowed to grow overnight. On day 3, exponentially growing cultures were subsequently diluted to OD_600_ = 0.25 and allowed to grow for 3 hr at 25°C. Temperature-sensitive alleles were then triggered by the addition of galactose to a 2% final concentration, the addition of doxycycline to a 50 μg/mL final concentration, and a temperature shift to 37°C. Where a release from an early metaphase arrest to a *cdc20-td*-mediated arrest was required, cells were first arrested with nocodazole; the nocodazole was then washed out and pellets were resuspended in at 37°C in 50 μg/mL doxycycline and either YEP (2% raffinose) or YEP (2% galactose). Cells remained in these conditions for 2 hr to ensure that passage to a *cdc20-td*-mediated arrest was attained. In experiments where Cdc20 was under control of the MET promoter, strains were cultured in minimal media lacking methionine −MET (2% raffinose), and Cdc20 inactivation was triggered by resuspension of the pellet in YEP (2% dextrose). For *TOP2* overexpression, exponentially growing cells in YEP (2% raffinose) were arrested in G_1_ by adding α factor, followed by release in YEP (2% glucose) or YEP (2% galactose) containing nocodazole (15 μg/mL). After 2 hr, cells reached G_2_/M. Samples were taken at that point and 2 hr later. Cells were kept arrested in nocodazole by the addition of a second dose of the drug 2 hr after the release from α factor.

### FACS Analysis

Approximately 1 × 10^7^ cells were sedimented at 9,500 rcf for 1 min, and pellets were resuspended and stored in 500 μL 70% ethanol at 4°C. Pellets were then resuspended in 250 μL 1× saline sodium citrate (SSC) supplemented with 0.1 mg/mL RNaseA and incubated at 37°C overnight. Then 50 μL 1× SSC supplemented with 0.75 mg/mL Proteinase K was added to the tubes, and cells were incubated at 50°C for 1 hr. A further 250 μL 1× SSC was added to the tubes, and samples were sonicated for 2 cycles (30 s on, 30 s off, low power) at 4°C. A total of 250 μL 1× SSC supplemented with 3 μg/mL propidium iodide was then added to the tubes and samples incubated at room temperature for 1 hr. Finally, 200 μL of each sample was read with a Becton Dickinson FACSCalibur, ensuring 20,000 events per sample.

### Genomic DNA Preparation

Cells were harvested at 4,000 rpm, and pellets were washed in ice-cold H_2_O and stored at −80°C. Pellets were resuspended in 11 mM Tris-HCl and 10 mM DTT and incubated on ice for 20 min. Cells were washed in ice-cold H_2_O, resuspended in spheroplasting buffer (1 M sorbitol, 50 mM Tris-HCL [pH 7.5], 1 mM CaCl_2_, 1 mM MgCl_2_, 14 mM β-mercaptoethanol, and 2,200 units Zymolyase T100), and incubated for 30 min at 37°C with shaking. Spheroplasts were sedimented in a fixed-angle rotor (Eppendorf F-34-6-38) at 6,000 rpm for 6 min, gently washed with 1 M sorbitol, transferred to 1.5 mL tubes, and sedimented for 1 min at 3,500 rcf at 4°C. Pellets were resuspended in 200 μL cold 0.4 M sorbitol, lysed on ice for 30 min by addition of 700 μL lysis buffer (25 mM HEPES/KOH [pH 8], 50 mM KCl, 10 mM MgSO_4_, 0.25% Triton X-100, 1 mM PMSF, 3 mM DTT, and protease inhibitor cocktail tablet [Roche]), and supplemented with 100 μg/mL RNaseA and 300 mM NaCl. Cell extracts were subsequently obtained by spinning the lysed spheroplasts at 12,000 rcf at 4°C for 5 min. Cleared lysates were loaded onto sucrose gradients prepared in a Biocomp gradient station and sedimented in a SW41 rotor (Beckman Optima L-100 XP Preparative Ultracentrifuge) at 18,000 rpm for 9 hr at 4°C. Gradients were fractionated into 30 equal fractions using a Biocomp gradient station.

### Southern Blot Analysis

Gradient fractions were separated on 0.5% agarose gels (0.5× TBE) containing 0.5 μg/mL ethidium bromide at 1.15 V/cm for 40 hr at 4°C. Gels were transferred to positively charged nylon transfer membranes (Hybond-N+, Amersham Biosciences). Blots were hybridized with fluorescein-labeled probes against plasmid-specific regions, and membranes were visualized following incubation with CDP-Star detection reagent for 10 min at room temperature. Three biological replicas were done for the Southern blot experiments. SCI were quantified using ImageJ software. The total amount of SCIs (including Cata, Catb, and Catc species) and monomers (supercoiled, relaxed, and linear species) was quantified. Graphs show the ratio between SCIs and monomers.

### Fluorescence Microscopy

A series of z-focal plane images were collected on Leica IRB using a Hamamatsu D742-95 digital camera and OpenLab software (Improvision). Images taken were phase, UV, and GFP filtered. To visualize the nuclei of intact cells, cells were resuspended in 1% Triton X-100 supplemented with DAPI.

### Sample Preparation and Western Blot Analysis

Samples were prepared by TCA extraction. Extracts were prepared as follows. Cells were collected by centrifugation (4,000 rpm, 2 min) and washed with 20% TCA. The TCA was aspirated and the pellets frozen at −80°C. All of the following purification steps were performed on ice with pre-chilled solutions. Cells were resuspended in 250 μL 20% TCA, glass beads were added, and the cells were broken by one 40 s cycle (power 5.5 in a FastPrep FP120 [BIO 101] machine). Tubes were pierced with a hot needle, placed onto fresh Eppendorfs, and spun (1,000 rpm, 2 min) to collect lysate minus glass beads. The glass beads were washed with 1 mL 5% TCA; this was added to the lysate and mixed by pipetting. The precipitated proteins were collected by centrifugation (14,000 rpm, 10 min, 4°C), and then pellets were washed with 750 μL 100% ethanol. Proteins were solubilized in 50 μL 1 M Tris (pH 8) and 100 μL ×2 SDS-PAGE loading buffer (60 mM Tris [pH 6.8], 2% SDS, 10% glycerol, and 0.2% bromophenol blue) and boiled for 5 min at 95°C. Insoluble material was removed by centrifugation (14,000 rpm, 5 min, room temperature), and the supernatant was either stored at −20°C or loaded immediately onto a SDS-PAGE minigel. Samples were either run on an 8% acrylamide gel in Tris-Glycine SDS or on running buffer using the Bio Rad Mini-PROTEAN 3 system. SDS-PAGE gels were transferred to polyvinylidene fluoride transfer membrane (Hybond-P, Amersham Biosciences) either in the Bio-Rad Mini Trans-Blot Electrophoretic Transfer Cell or by using the XCell SureLock Mini Cell Transfer module (Invitrogen). The Bio-Rad system was used in conjunction with Tris-Glycine blotting buffer (National Diagnostics) containing 20% methanol and run for 1 hr at 200 V or overnight at 30 V. Membranes were blocked in 5% skimmed milk powder in PBS with 0.1% Tween 20 (PBS-T) for 1 hr or overnight at 4°C, then incubated with anti-Rad53 antibody 12CA5 at a 1/5,000 dilution in blocking solution for between 1 hr at room temperature to overnight at 4°C. Following several washes in PBS-T, membranes were incubated with the sheep anti-rabbit IgG horseradish-peroxidase-linked antibody (GE Healthcare) at a 1/10,000 dilution in blocking solution. After several further washes in PBS-T, membranes were incubated with the ECL Plus Western Blotting Detection System (GE Healthcare) followed by exposure to ECL Hyperfilm (GE Healthcare) to detect the secondary antibody.

## Author Contributions

N.S. and L.A. conceived the study. N.S. performed most experiments. J.L. and R.T. performed minichromosome topology assays. J.G.-L. and G.P.-M. performed experiments for cell-cycle analyses of *TOP2* overexpression. L.A. wrote the manuscript with input from all authors.

## Supplemental Information

Document S1. Figures S1–S3

Document S2. Article plus Supplemental Information

## References

[bib1] Baldi M.I., Benedetti P., Mattoccia E., Tocchini-Valentini G.P. (1980). In vitro catenation and decatenation of DNA and a novel eucaryotic ATP-dependent topoisomerase. Cell.

[bib2] Baxter J., Aragón L. (2012). A model for chromosome condensation based on the interplay between condensin and topoisomerase II. Trends Genet..

[bib3] Baxter J., Sen N., Martínez V.L., De Carandini M.E., Schvartzman J.B., Diffley J.F., Aragón L. (2011). Positive supercoiling of mitotic DNA drives decatenation by topoisomerase II in eukaryotes. Science.

[bib4] Bazett-Jones D.P., Kimura K., Hirano T. (2002). Efficient supercoiling of DNA by a single condensin complex as revealed by electron spectroscopic imaging. Mol. Cell.

[bib5] Champoux J.J., McCoubrey W.K., Been M.D. (1984). DNA structural features that lead to strand breakage by eukaryotic type-I topoisomerase. Cold Spring Harb. Symp. Quant. Biol..

[bib6] Charbin A., Bouchoux C., Uhlmann F. (2014). Condensin aids sister chromatid decatenation by topoisomerase II. Nucleic Acids Res..

[bib7] Díaz-Martínez L.A., Giménez-Abián J.F., Clarke D.J. (2008). Chromosome cohesion—rings, knots, orcs and fellowship. J. Cell Sci..

[bib8] Farcas A.M., Uluocak P., Helmhart W., Nasmyth K. (2011). Cohesin’s concatenation of sister DNAs maintains their intertwining. Mol. Cell.

[bib9] Giménez-Abián J.F., Clarke D.J., Mullinger A.M., Downes C.S., Johnson R.T. (1995). A postprophase topoisomerase II-dependent chromatid core separation step in the formation of metaphase chromosomes. J. Cell Biol..

[bib10] Guacci V., Koshland D., Strunnikov A. (1997). A direct link between sister chromatid cohesion and chromosome condensation revealed through the analysis of MCD1 in S. cerevisiae. Cell.

[bib11] Hirano T. (2005). SMC proteins and chromosome mechanics: from bacteria to humans. Philos. Trans. R. Soc. Lond. B Biol. Sci..

[bib12] Hirano T. (2012). Condensins: universal organizers of chromosomes with diverse functions. Genes Dev..

[bib13] Kimura K., Hirano T. (1997). ATP-dependent positive supercoiling of DNA by 13S condensin: a biochemical implication for chromosome condensation. Cell.

[bib14] Koshland D., Hartwell L.H. (1987). The structure of sister minichromosome DNA before anaphase in Saccharomyces cerevisiae. Science.

[bib15] Koshland D., Strunnikov A. (1996). Mitotic chromosome condensation. Annu. Rev. Cell Dev. Biol..

[bib16] Leonard J., Sen N., Torres R., Sutani T., Jarmuz A., Shirahige K., Aragón L. (2015). Condensin relocalization from centromeres to chromosome arms promotes Top2 recruitment during anaphase. Cell Rep..

[bib17] Losada A., Hirano T. (2001). Intermolecular DNA interactions stimulated by the cohesin complex in vitro: implications for sister chromatid cohesion. Curr. Biol..

[bib18] Losada A., Hirano M., Hirano T. (1998). Identification of Xenopus SMC protein complexes required for sister chromatid cohesion. Genes Dev..

[bib19] Losada A., Hirano M., Hirano T. (2002). Cohesin release is required for sister chromatid resolution, but not for condensin-mediated compaction, at the onset of mitosis. Genes Dev..

[bib20] Maxwell, J. (1871), reprint (2001). Theory of Heat (Dover).

[bib21] Michaelis C., Ciosk R., Nasmyth K. (1997). Cohesins: chromosomal proteins that prevent premature separation of sister chromatids. Cell.

[bib22] Murray A.W., Szostak J.W. (1985). Chromosome segregation in mitosis and meiosis. Annu. Rev. Cell Biol..

[bib23] Nasmyth K. (2001). Disseminating the genome: joining, resolving, and separating sister chromatids during mitosis and meiosis. Annu. Rev. Genet..

[bib24] Nitiss J.L., Beck W.T. (1996). Antitopoisomerase drug action and resistance. Eur. J. Cancer.

[bib25] O’Malley F.P., Chia S., Tu D., Shepherd L.E., Levine M.N., Huntsman D., Bramwell V.H., Andrulis I.L., Pritchard K.I. (2011). Topoisomerase II alpha protein and responsiveness of breast cancer to adjuvant chemotherapy with CEF compared to CMF in the NCIC CTG randomized MA.5 adjuvant trial. Breast Cancer Res. Treat..

[bib26] Postow L., Crisona N.J., Peter B.J., Hardy C.D., Cozzarelli N.R. (2001). Topological challenges to DNA replication: conformations at the fork. Proc. Natl. Acad. Sci. USA.

[bib27] Pulleyblank D.E. (1997). Of topo and Maxwell’s dream. Science.

[bib28] Rybenkov V.V., Ullsperger C., Vologodskii A.V., Cozzarelli N.R. (1997). Simplification of DNA topology below equilibrium values by type II topoisomerases. Science.

[bib29] St-Pierre J., Douziech M., Bazile F., Pascariu M., Bonneil E., Sauvé V., Ratsima H., D’Amours D. (2009). Polo kinase regulates mitotic chromosome condensation by hyperactivation of condensin DNA supercoiling activity. Mol. Cell.

[bib30] Stone M.D., Bryant Z., Crisona N.J., Smith S.B., Vologodskii A., Bustamante C., Cozzarelli N.R. (2003). Chirality sensing by Escherichia coli topoisomerase IV and the mechanism of type II topoisomerases. Proc. Natl. Acad. Sci. USA.

[bib31] Straight A.F., Belmont A.S., Robinett C.C., Murray A.W. (1996). GFP tagging of budding yeast chromosomes reveals that protein-protein interactions can mediate sister chromatid cohesion. Curr. Biol..

[bib32] Stuchinskaya T., Mitchenall L.A., Schoeffler A.J., Corbett K.D., Berger J.M., Bates A.D., Maxwell A. (2009). How do type II topoisomerases use ATP hydrolysis to simplify DNA topology beyond equilibrium? Investigating the relaxation reaction of nonsupercoiling type II topoisomerases. J. Mol. Biol..

[bib33] Sundin O., Varshavsky A. (1980). Terminal stages of SV40 DNA replication proceed via multiply intertwined catenated dimers. Cell.

[bib34] Timsit Y., Várnai P. (2010). Helical chirality: a link between local interactions and global topology in DNA. PLoS ONE.

[bib35] Vosberg H.P. (1985). DNA topoisomerases: enzymes that control DNA conformation. Curr. Top. Microbiol. Immunol..

[bib36] Wang J.C. (1996). DNA topoisomerases. Annu. Rev. Biochem..

[bib37] Worland S.T., Wang J.C. (1989). Inducible overexpression, purification, and active site mapping of DNA topoisomerase II from the yeast Saccharomyces cerevisiae. J. Biol. Chem..

