# Supplementary material for: Physical Proximity of Sister Chromatids Promotes Top2-Dependent Intertwining
Source: Mol Cell. 2016 Oct 6;64(1):134–47. doi: 10.1016/j.molcel.2016.09.007 (PMC5065527; doi:10.1016/j.molcel.2016.09.007)
Supplement: Document S1. Figures S1–S3 [file mmc1.pdf]

Molecular Cell, Volume *64*

Supplemental Information

Physical Proximity of Sister Chromatids Promotes Top2-Dependent Intertwining

Nicholas Sen, Joanne Leonard, Raul Torres, Jonay Garcia-Luis, Gloria Palou-Marin, and Luis Aragón

Figure S1

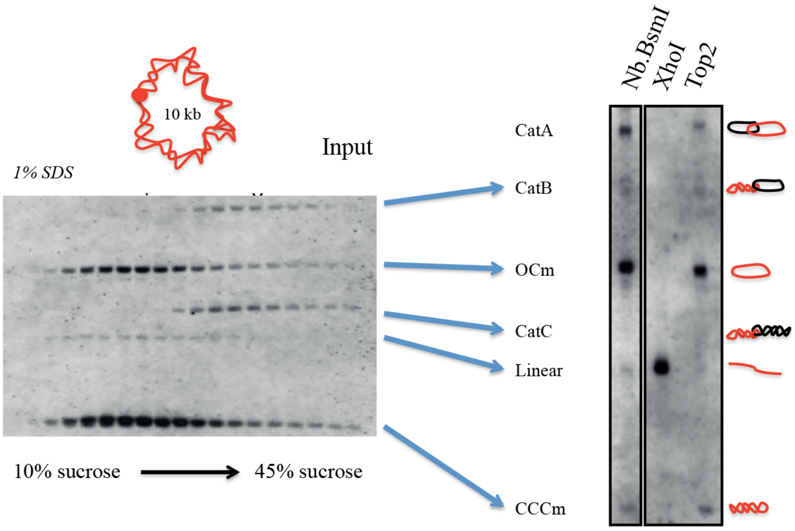

10 kb
Input
1% SDS
10% sucrose
45% sucrose
CatA
CatB
OCm
CatC
Linear
CCCm
Nb.BsmI
XhoI
Top2

Figure S2

Figure 1

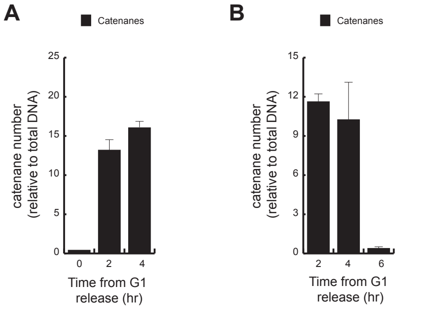

Figure 2

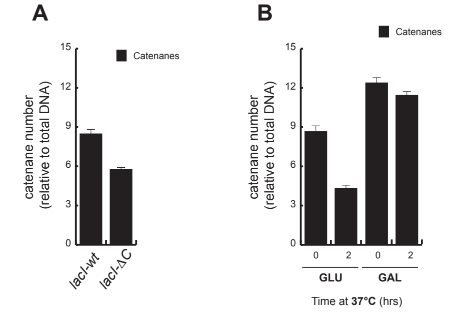

Figure 3

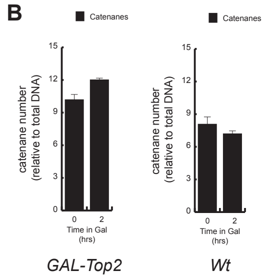

Figure 5

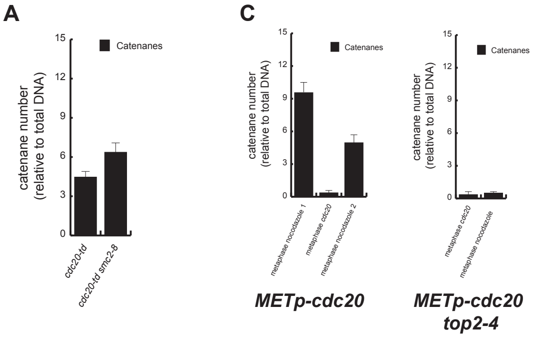

Figure S3

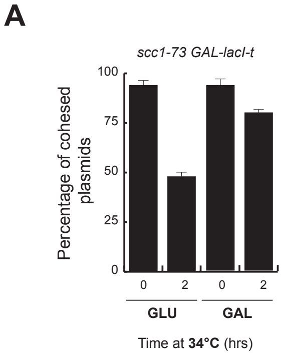
A
scc1-73 GAL-lacI-t
Percentage of cohesed plasmids
100
75
50
25
0
0
2
0
2
GLU
GAL
Time at 34°C (hrs)

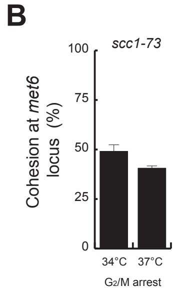
B
scc1-73
Cohesion at met6 locus (%)
100
75
50
25
0
34°C
37°C
G2/M arrest

Supplementary Figure Legends

Supplementary Figure S1

Identification by restriction enzyme treatment of plasmid species from sucrose gradient fractions. DNA from fractions collected from metaphase arrested cells (nocodazole arrests) was pooled together and treated with either nicking enzyme *Nb.BsmI*, restriction endonuclease *XhoI* or recombinant topoisomerase II protein (Top2). Treatment with Nt.BsmI caused relaxation of monomers (OCm) and relaxation of dimers (CatA). Digestion with *XhoI* led to accumulation of linear monomers (Linear). Treatment with Top2 resulted in relaxation of supercoiled monomers and decatenation of dimer species, consequently supercoiled monomers (OCCm) and catenated dimers (CatA, CatB and CatC species) were reduced and relaxed monomers accumulated (OCm). From these three treatments we were able to identify plasmid species in the gradient fractions. Note that *Nb.BsmI* treated samples were run on a separate gel from *XhoI* and Top2 treated samples.

Supplementary Figure S2

Quantification of catenanes (SCIs) (including a-, b- and c-type) in the experiments shown in the article figures relative to total DNA. The signals were quantified using ImageJ software and the catenanes were quantified relative to all the DNA signals in the blots. Graphs show the mean ± s.d. from three independent experiments. The corresponding figure numbers and panels are indicated for each graph.

Supplementary Figure S3

A. *scc1-73* strain bearing the 10kb-circular minichromosome containing the lacO array and the *lacI-t GFP* construct under the galactose promoter was grown in raffinose media and synchronised in G1. The culture was divided in two. One half was released in galactose media (expressing *lacI-t*) while the other half was released in glucose media (repressing *lacI-t*). Both cultures were released from the G1 arrest in the presence of nocodazole at 25°C, and upon metaphase arrest, the cultures were shifted to 34°C to inactivate *scc1-*

73. Samples were scored for plasmid cohesion at the metaphase block at 25°C and two hours after temperature shift. The percentage of cells with cohesed (single dots) plasmids is shown. Error bars indicate the mean ± s.d. from three independent experiments). **B.** *scc1-73* strain carrying a lacO array inserted at the met6 locus in chromosome V and a *lacI-GFP* construct was synchronised in G1 at 25°C and released in the presence of nocodazole at 34°C or 37°C inactivate *scc1-73*. Loss of sister chromatid cohesion was scored at the metaphase two hours after temperature shift. The percentage of cells with cohesed (single dots) dots is shown. Error bars indicate the mean ± s.d. from three independent experiments).
